# Dynamic interactions between anterior insula and anterior cingulate cortex link perceptual features and heart rate variability during movie viewing

**DOI:** 10.1162/netn_a_00295

**Published:** 2023-06-30

**Authors:** Saurabh Sonkusare, Katharina Wegner, Catie Chang, Sasha Dionisio, Michael Breakspear, Luca Cocchi

**Affiliations:** Department of Psychiatry, University of Cambridge, Cambridge, United Kingdom; QIMR Berghofer Medical Research Institute, Brisbane, Australia; Ghent University, Ghent, Belgium; Vanderbilt University, USA; The University of Queensland, Brisbane, Australia; Advanced Epilepsy Unit, Mater Centre for Neurosciences, Mater Hospitals, Brisbane, Australia; The University of Newcastle, Newcastle, Australia

**Keywords:** Connectivity, Neural dynamics, Movie, Heart rate, DCM, Emotions

## Abstract

The dynamic integration of sensory and bodily signals is central to adaptive behaviour. Although the anterior cingulate cortex (ACC) and the anterior insular cortex (AIC) play key roles in this process, their context-dependent dynamic interactions remain unclear. Here, we studied the spectral features and interplay of these two brain regions using high-fidelity intracranial-EEG recordings from five patients (ACC: 13 contacts, AIC: 14 contacts) acquired during movie viewing with validation analyses performed on an independent resting intracranial-EEG dataset. ACC and AIC both showed a power peak and positive functional connectivity in the gamma (30–35 Hz) frequency while this power peak was absent in the resting data. We then used a neurobiologically informed computational model investigating dynamic effective connectivity asking how it linked to the movie’s perceptual (visual, audio) features and the viewer’s heart rate variability (HRV). Exteroceptive features related to effective connectivity of ACC highlighting its crucial role in processing ongoing sensory information. AIC connectivity was related to HRV and audio emphasising its core role in dynamically linking sensory and bodily signals. Our findings provide new evidence for complementary, yet dissociable, roles of neural dynamics between the ACC and the AIC in supporting brain-body interactions during an emotional experience.

## Author Summary

Naturalistic stimulus such as movies provide an optimal platform to study time-resolved neural interactions supporting integration of sensory and bodily information. Here, we obtain intracranial recordings from anterior insular cortex and the anterior cingulate cortex while participants (undergoing clinical evaluation for epilepsy) viewed a short movie capable of eliciting robust physiological responses. We first characterise the spectral properties with subsequent model driven approach to probe moment-to-moment neural interactions between the anterior insular cortex and the anterior cingulate cortex. Our findings highlight how this neural system plays a key role in linking the contextual integration of sensory information with that of internal body status.

## INTRODUCTION

Brain activity continuously adapts to sensory inputs, triggering physiological responses to threat and emotionally salient stimuli (Critchley, 2005; Damasio et al., 2000; Menon, 2011). The bidirectional coupling between external sensory cues and bodily physiological adaptations represents a key function of the central nervous system. However, the dynamic neural processes supporting the integration of multimodal sensory and cognitive-emotional information with somatic responses remain poorly understood.

The salience network, anatomically anchored in the dorsal anterior cingulate cortex (ACC) and anterior insular cortex (AIC) (Seeley et al., 2007; Touroutoglou et al., 2012, 2016), has been consistently implicated in the synthesis and integration of signals from the external environment and the body (Critchley, 2005; Critchley et al., 2005; Seth, 2013; Uddin, 2015). Previous research suggests interoceptive signals from various bodily afferents are processed by the posterior insula while the integration of high-level physiological and cognitive functions occur anteriorly (Craig, 2009; Critchley, 2005; Critchley et al., 2005; Farb et al., 2013; Kucyi & Parvizi, 2020; Nguyen et al., 2016; Simmons et al., 2013; Tian & Zalesky, 2018). The ACC is believed to receive and integrate multisensory perceptual information and underpins diverse cognitive functions, including emotion, motivation, and error monitoring (Ito et al., 2003; Ullsperger & von Cramon, 2001; Weston, 2012).

Unified accounts of the function of the ACC-AIC circuit suggest that the ACC supports the cognitive appraisal of sensory information and the modulation of interoceptive representations in the AIC (Bush et al., 2000; Hall et al., 2022; Harrison et al., 2015; Weston, 2012). The integration of multimodal information is especially relevant in emotional contexts which induce changes in peripheral body signals such as heart rate (HR), respiration, and perspiration. Furthermore, facial expressions appear to evoke activity in these regions, which are specific to the emotion expressed, as observed in intracranial-EEG recordings (iEEG) (Bijanzadeh et al., 2022). Functional magnetic resonance imaging (fMRI) studies using peripheral physiological responses such as HR and skin conductance have demonstrated the role of the AIC-ACC dyad in interoception (Gray et al., 2009; Nagai et al., 2004). Analyses of fMRI data have also suggested that slow fluctuations (∼30 sec) in functional connectivity between the ACC and the whole-brain covary with heart rate variability (HRV) (Chang et al., 2013). However, the limitations of fMRI prohibit analyses of rapid (<1 sec) neural computations underpinning brain-body communication. High-fidelity recordings could overcome these challenges thus unravelling the nature of the neural coupling between the ACC and the AIC supporting the dynamic process of perceptual and body signals.

To disentangle the functional relevance of dynamic patterns of interactions between the AIC and the ACC, we used iEEG depth recordings. This invasive measure of neural activity has excellent spatiotemporal resolution compared to other neuroimaging techniques, including fMRI. Moreover, iEEG is minimally affected by cardiorespiratory and motion artefacts (Parvizi & Kastner, 2018). We acquired neural recordings from the ACC and the AIC while surgical participants (suffering from epilepsy) watched an emotionally salient movie. This allowed capturing not only the induced neural dynamics but also the accompanying physiological response of HRV evoked during an ecologically valid sensory experience when compared to the traditional task paradigms utilised in neuroimaging studies such as abstract and static images (Sonkusare et al., 2019a, 2019b; but see Grall & Finn, 2022).

Using these unique data, we first sought to understand power spectral features associated with movie viewing as well as functional connectivity between ACC and AIC. Furthermore, we wanted to characterise the time-resolved changes in feedforward and feedback interactions in ACC-AIC cortical circuit, that is, effective connectivity. This objective mandates inference regarding an underlying generative model (Friston, 2011). We resolved context-specific dynamic neural interactions between ACC and AIC using a validated biophysical model: dynamic causal modelling (DCM) for cross-spectral density (CSD), which uses power spectrum as the data feature with an underlying neuronal model of canonical microcircuit (CMC). The adopted biophysical model is also uniquely positioned to address our question, as it incorporates neuroanatomical parameters supporting ascending (bottom-up/feedforward) and descending (top-down/feedback) connections (Barbas & Rempel-Clower, 1997; Felleman & Van Essen, 1991; Hilgetag et al., 2000). In addition, although DCM has been traditionally used for noninvasive datasets where the use of a forward (observation) model is necessary, the core feature of DCM is the underlying biophysical (generative) model, here the CMC model. In the presence of invasive and more direct recordings—such as those we use—a generative model remains an advantageous feature of effective connectivity inference.

To address how the ACC-AIC circuit integrates sensory-perceptual and bodily responses, we studied the temporal concordance between time-resolved parameters of dynamic effective connectivity and their association to low- (audio, luminance) and high- (salience, emotion) level movie features. Bodily response fluctuations were investigated by measuring changes in frequency domain HRV features. HRV results from changes in interbeat interval and is a typical marker of autonomic activity (Ravenswaaij-Arts et al., 1993). Previous work has also shown that cognitive and emotionally salient contexts can influence the HRV via parasympathetic and sympathetic mechanisms (Jönsson & Sonnby-Borgström, 2003; Wallentin et al., 2011). Thus, with a concurrent assessment of sensory stimulus properties, changes in HRV, local neural activity and their coupling, we investigated how the ACC-AIC circuit leads to coherent integration of sensory information and peripheral bodily responses.

## MATERIALS AND METHODS

### Movie Viewing Data

#### Participants.

Participants were selected from a cohort of 13 individuals with intractable epilepsy, implanted with stereotactic depth electrodes for clinical evaluation at Mater Advanced Epilepsy Unit (Brisbane, Australia). These 13 were all consenting participants acquired throughout the available study period and are typical for single-site intra-operative data using specific cognitive paradigms. Informed consent was obtained from all participants. The placement of electrodes was determined exclusively by clinical criteria. Inclusion criteria for the present study were (i) location of electrodes within both the ACC and the AIC, (ii) epileptogenic zone outside the ACC, and (iii) the AIC ability to provide written consent. Five individuals met these criteria. Patient characteristics are provided in Table 1. The study was approved by the Human Research Ethics Committee of the Mater Hospital and the QIMR Berghofer Medical Research Institute and performed in accordance with the Declaration of Helsinki.

**Table 1.** Patient profile

Patient ID	Sex	Age (Y)	Handedness	Sz onset age	Diagnosed epileptogenic zone
P1	F	16	R	8	Right amygdala, hippocampus, and left anterior cingulate
P2	M	56	R	17	Right orbitofrontal region
P3	F	26	R	15	Right posterior middle temporal gyrus, posterior superior temporal gyrus, supramarginal gyrus, amygdala, posterior insula
P4	M	27	R	10	Left anterior long gyrus of insula
P5	M	47	R	33	Left mesial temporal pole

#### Stereo-EEG recordings.

The implantation of intracranial depth multicontact electrodes (DIXI, 10–15 contacts, electrode diameter: 1 mm, intercontact spacing 1.5 mm) was performed by adopting a stereotactic procedure. Electrodes were implanted in each subject based on the likely seizure onset zone inferred by the epileptologist (author S. D.) using noninvasive preoperative indices. Stereo-EEG signals sampled at 1 kHz were recorded on a Neurofax EEG-1200 system (Nihon Koden, Japan). All experimental task data were processed off-line using EEGLAB (Delorme & Makeig, 2004) and custom MATLAB (MathWorks) scripts. Data were also visually inspected. The signals were downsampled to 500 Hz and band-passed filtered between 0.1 to 195 Hz using a zero-phase lag filter (FIR) (Delorme & Makeig, 2004). Data were rereferenced with bipolar referencing scheme. Bipolar referencing has been demonstrated as advantageous since it removes contamination from activity at the online reference electrode and highlights activity that is local (Lachaux et al., 2003; Shirhatti et al., 2016). Our preprocessing strategy is also consistent with analyses adopted in other iEEG studies employing patients with epilepsy (Jerbi et al., 2009; Lachaux et al., 2003; Michelmann et al., 2018; Palva et al., 2005; Potes et al., 2012).

#### Electrode localization.

**Figure 1.**
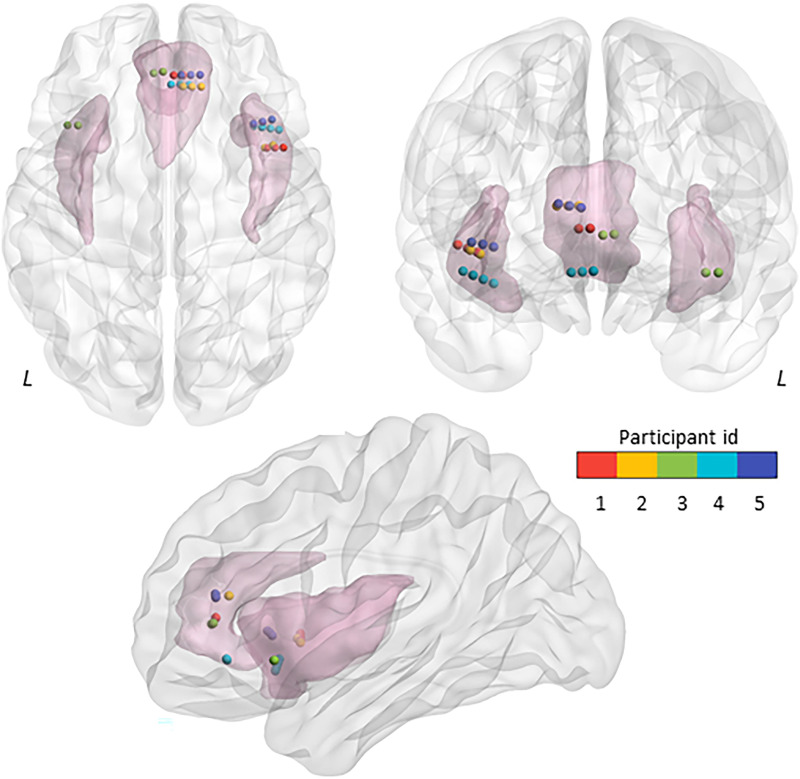
Electrode channel locations for the movie viewing dataset. Anatomical locations of the electrode channels implanted in the left and right anterior insular cortex (AIC, 14 contacts across subjects) and the anterior cingulate cortex (ACC, 13 contacts) are shown in MNI space. L = Left.

Intracranial electrodes were localized on a 3-D Montreal Neurological Institute (MNI) brain template using a fusion of preoperative magnetic resonance (MR) and postoperative computed tomography (CT) scans using Brainstorm (Tadel et al., 2011). We started by coregistering the CT image to MR T1 image and normalising them to the MNI template. The coregistered and normalized CT and MR images were then visually inspected for the anatomical location of the electrode channels. The electrodes channels were also assessed on the AAL atlas (Tzourio-Mazoyer et al., 2002) and visually inspected to confirm their location in the anatomical regions of interest. The location of the electrodes in MNI space was corroborated against implantation maps generated by an epileptologist (S. D.) to clinically localize seizures. Four participants with right AIC (anterior short gyrus) and right ACC (cingulate gyrus) electrode channels and one patient with left AIC and left ACC met our inclusion criteria (see above). In all, we had 14 contacts in the AIC and 13 contacts in the ACC from five participants (Table 2 and Figure 1). Electrode contacts were visualised on a MNI brain template using BrainNet Viewer (Xia et al., 2013).

**Table 2.** MNI coordinates

Participant ID	AIC MNI coordinats (x, y, z)	ACC MNI coordinats (x, y, z)
P1	40.4, 9.7, −0.5	6.0, 39.6, 7.6
	43.7, 9.8, 0.9	2.1, 39.6, 7.7
	47.1, 9.8, 2.1	
P2	39.5, 9.8, −1.1	5.8, 35.0, 15.4
	42.9, 10.8, 0.4	9.6, 35.0, 15.4
		13.4, 34.9, 15.4
P3	−37.2, 19.0, −7.4	−6.1, 40.5, 5.7
	−41.5, 19.1, −7.4	−2.3, 41.1, 5.4
P4	34.9, 18.7, −11.1	8.5, 35.6, −7.3
	38.4, 18.2, −9.7	4.8, 35.7, −7.1
	41.9, 17.7, −8.3	1.0, 35.8, −6.9
	45.3, 17.2, −6.9	
P5	34.9, 19.7, 1.4	5.2, 39.6, 14.8
	38.6, 20.5, 2.3	9.1, 39.7, 15.4
	42.4, 21.4, 3.0	12.9, 39.8, 16.2

#### Experimental paradigm.

**Figure 2.**
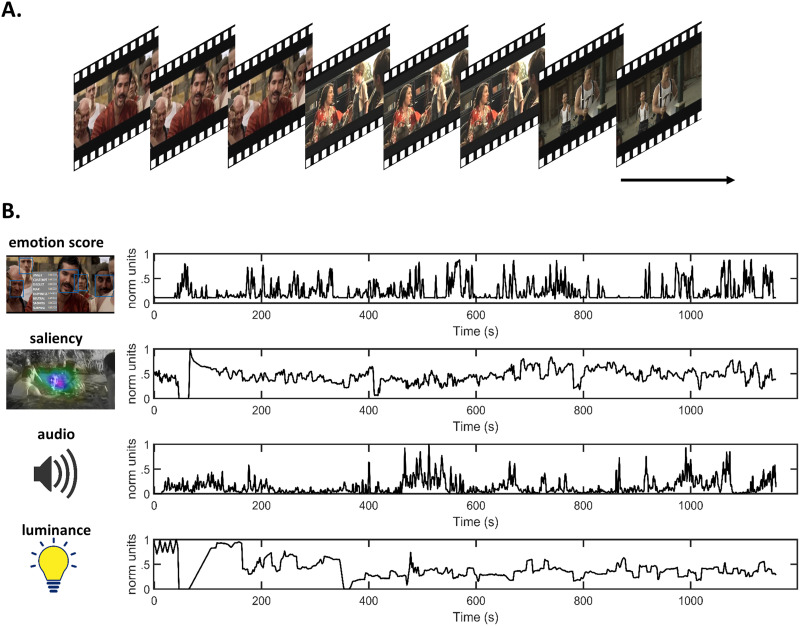
Movie task and features. (A) A continuous short movie of 20-min duration was used as stimulus. (B) Time series associated with movies features averaged for 1-sec windows.

Participants viewed “The Butterfly Circus” (Weigel et al., 2009), a short movie with a duration of 1,155 seconds that has previously been used as a stimulus in our earlier fMRI studies, which also provides thematic content and details of the movie (Meer et al., 2020; Nguyen et al., 2017) (Figure 2). Briefly, the adopted stimuli is a dramatic emotional film that narrates the story of a man born without limbs who overcomes obstacles and finds self-worth and purpose with the help of a showman of a renowned circus. A countdown of 10 seconds was prefixed to the movie for participants to attend to before the start of the movie. The movie was presented using a 14-inch laptop LCD screen (1,366 × 768, refresh rate of 60 Hz) placed at approximately 60 cm away from the subject. The movie stimulus was delivered using Presentation software (Version 18.0, Neurobehavioral Systems, Inc., Berkeley, CA, www.neurobs.com).

#### Movie features extracted for investigating exteroception.

The movie audio, luminance, facial emotional expressions, and visual salience characteristics were extracted to represent indices of exteroceptive information (Figure 2). Audio information was estimated using the Hilbert transform of the audio signal. Pliers toolbox (https://github.com/tyarkoni/pliers) was used to extract luminance and salience properties (McNamara et al., 2017) as employed in a previous study (Ren et al., 2018). Luminance was computed as the average luminosity across the pixels. Visual saliency was defined by validated computer vision criteria, allowing the automatic detection of the conspicuous objects in a scene (Li et al., 2018). Movie frames were processed using the Microsoft API emotion recognition and scoring software (Del Sole, 2018). This software detects faces in each frame and returned a score for the following emotions: anger, contempt, disgust, fear, happiness, neutral, sadness, surprise, and neutral. For movie frames without faces, a score of zero was returned. Emotion scores averaged for each second were ranked in descending order, with the overall total score summing to 1. To define the emotional load of each second, we used the sum of all emotion scores, excluding neutral. All movie features except emotion (which already had a range of 0–1) were individually rescaled to have values between 0–1. Movie features can share temporal patterns: for example, sad emotional scenes are usually depicted with low audio intensity. Hence, movie features were orthogonalised (spm_orth.m) (Penny et al., 2011) before using them for further analysis. Specifically, emotion scores were inputted as the first modulator with salience, audio, and luminance added as subsequent modulators. The order of these modulators was chosen prior to any analyses and informed by the likelihood of the features to capture significant variance of relevance to our hypotheses.

#### Heart rate variability.

ECG signals were recorded using a Nihon Kohden system (https://www.nihonkohden.com/) with the lead placed on the chest or the back, and the signals were recorded at the sampling rate of 1000 Hz. ECG data from one subject (P5) was not recorded. Heartbeats (R peaks) were first detected automatically using the peak detection algorithm implemented in QRSTool software (Allen et al., 2007). The detected R peaks were visually checked, and the misidentified ones were manually corrected. The timing of R peaks was noted and was subsequently used to compute HRV frequency domain measures using HRVAS toolbox (Ramshur, 2010). These were calculated on the whole movie data of 20 min via the auto-regressive method using a window size of 16 sec, with 15 samples overlap, nonequispaced fast Fourier transform of 1,024 and cubic spline interpolation rate of 2 Hz as used in our earlier studies. Specifically, using these parameters we were able to show instantaneous high-frequency (HF) and Lower frequency (LF) HRV fluctuations induced by a loud sound akin to a startle reflex (Sonkusare et al., 2019a, 2019b). HRV data metrics were computed for the whole stimulus but edge effects for frequency estimation required the deletion of approximately 9 sec of data at the beginning and the end of each recording. These resulting outputs were then epoched corresponding to iEEG analyses. Time-frequency decompositions of interbeat intervals are typically linked to autonomic influences in distinct frequency bands. LF HRV (0.04–0.14 Hz) mainly reflects changes in sympathetic and parasympathetic outflows, while HF variability (0.15 to 0.4 Hz) is primarily due to modulation of parasympathetic outflow (Xhyheri et al., 2012). HF HRV power is also coupled to respiration, reflecting the respiratory sinus arrhythmia thought to arise from the modulation of vagal activity during respiration (Camm, 1996; although see Thomas et al., 2019). However, respiration is also affected by emotions (Boiten et al., 1994). Hence, we used the LF/HF ratio as a proxy of sympatho-vagal balance (Pagani et al., 1984, 1986; but see Billman, 2013; Shaffer et al., 2014). HRV also shows a substantial diurnal variation. Our data were acquired in a surgical setting, precluding the planning and formal analysis of the potential impact of these daily fluctuations on our results.

#### Resting-state data.

We benchmarked our analyses of these movie viewing data against an open access resting-state iEEG dataset. This dataset consists of preprocessed curated artifact-free iEEG data from 106 participants acquired in an “eyes closed” resting wakefulness state obtained from an open source data repository shared by Gotman and colleagues at the MNI (Frauscher et al., 2018; MNI Open iEEG Atlas—Document Repository, https://mni-open-ieegatlas.research.mcgill.ca). The data and preprocessing details are described elsewhere (Das & Menon, 2020). Briefly, preprocessing iEEG signals were band-pass filtered at 0.5–80 Hz, downsampled to 200 Hz, line noise was removed, and time series from each electrode was z-normalized by removing mean and scaling by the standard deviation. We found six participants (from the 106 participants) with implants both in ACC and AIC (P6, P30, P50, P60, P76, P106). In total, there were 13 contacts in AIC and 9 in ACC. Visual inspection of iEEG data revealed two subjects with flat recordings for 2 sec; for P6, 18–20 sec; and for P76, 57–59 sec. Hence, we only used 30 sec of continuous data from all these participants and deleted the first 20 and final 10 sec of data. The details of patient demographics and the MNI coordinates of the ACC and AIC contacts are provided in the Supporting Information (Table S1).

### Data Analysis

#### Power spectrum.

Power spectra for movie data and resting data were computed using a sliding Welch window of 2 sec, overlap of 1 sec with frequency resolution of .25 Hz. The signal’s power was computed for each contact and plotted. We used Fitting Oscillations and One-Over-F (FOOOF) algorithm (Donoghue et al., 2020) applied to 2–45 Hz power spectra to accurately separate the LFP power spectral densities into aperiodic (1/f-like component) and periodic oscillatory components modelled as Gaussian peaks.

#### Dynamic causal modelling.

**Figure 3.**
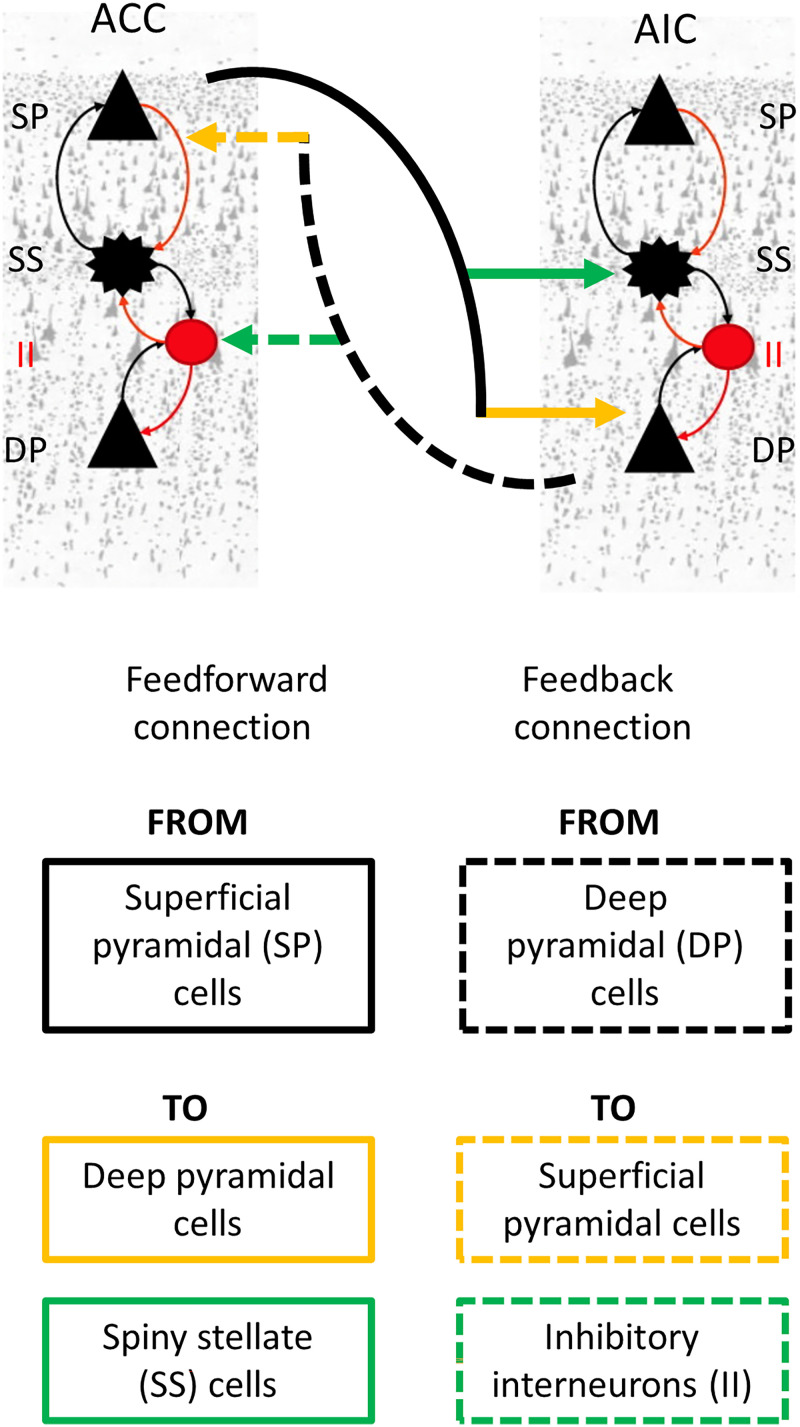
Simplified canonical microcircuit model. Each neuronal population, for example, anterior cingulate cortex (ACC) and anterior insular cortex, has four distinct neuronal subpopulations: (i) spiny stellate cells, (ii) superficial pyramidal cells, (iii) inhibitory interneurons, and (iv) deep pyramidal cells. Feedforward and the feedback connections form the interconnections between two neuronal populations differing in their origin and termination. Feedforward and feedback connections are only shown for ACC. Equivalent and corresponding connections exist for AIC. Neuronal cells shown are adapted from (Moran et al., 2013).

DCM provides a formal framework to infer causal (effective) connectivity between brain regions from neurophysiological data (Friston et al., 2003). In this study, we used DCM implementing the CMC biophysical model (Figure 3) (Auksztulewicz & Friston, 2015). In this cyto-architecturally inspired model, each neural mass (local population) is composed of four distinct neuronal subpopulations: (i) spiny stellate cells, (ii) superficial pyramidal cells, (iii) inhibitory interneurons, and (iv) deep pyramidal cells (Pinotsis et al., 2012). The interconnections between these cellular subpopulations differ in terms of their origin and termination. Self-connections are referred to as intrinsic connections and those between-regions are defined as extrinsic connections. Extrinsic connections are further divided into three types (Felleman & Van Essen, 1991): (i) feedforward connections; (ii) feedback connections; and (iii) lateral connections (Felleman & Van Essen, 1991). Feedforward connections originate at the superficial pyramidal cell layer and terminate either at the deep pyramidal layer or at the spiny stellate cell layer. Similarly, feedback connections originate at the deep pyramidal cell layer and terminate either at the superficial pyramidal layer or at the inhibitory interneuron cell layer. Lateral connections involve all cellular layers linking brain regions at an equivalent level of the cortical hierarchy. Here, the AIC-ACC patterns of connectivity were modelled using only feedforward (two for each region) and feedback (two for each region) connections. DCM estimation thus yielded eight connectivity parameters (two feedforward and two feedback connections for each node).

Given prior evidence for strong bidirectional anatomical connections between the ACC and the AIC (van den Heuvel et al., 2015; Von Economo, 2009), we employed a fully connected model. To obtain the data features for model inversion (i.e., fitting the model to the empirical data to output effective connectivity estimates), we implemented DCM for CSD (Friston et al., 2012). This DCM implementation uses real and imaginary parts of the cross-spectra (Fourier transform of the cross-correlation function) computed between the neuronal recordings as data features (Friston et al., 2012). We used the standard frequency range (2–45 Hz), known to support a broad range of sensory processes, emotions, and interoceptive functions (Luck, 2014; Wang, 2010). The effective connectivity parameters were estimated independently for each 1-sec window for all possible combinations of channel pairs from each subject. There were 37 channel pairs from the movie data and 21 channel pairs from the rest data. Overall, 42,735 model inversions for movie data and 630 for rest data were undertaken. To characterise the time-resolved (between-window) and intersubject consistency of the connectivity parameters estimated via DCM, we used a multilevel parametric empirical Bayes (PEB) approach (Van de Steen et al., 2019). PEB provides several advantages in a study with small sample size and several time-varying connectivity estimates. This technique takes into account both the estimates of peak mean connectivity as well as their covariance. The influence of parameter estimates showing high consistency across the group is up-weighted while noisy parameters’ estimates are down-weighted (Zeidman et al., 2019). The PEB approach adopted herein involves the following:

1. *Between-window effects*. Accommodating temporal fluctuations in the DCM model parameters involves assumptions regarding the window-to-window smoothness and the overall longer term trends. Similar to estimating DCMs, these are captured by a small number of temporal basis functions. A Bayesian general linear model approach was thus used to describe time variability in the connectivity parameters as the weighted average of a number of such smooth regressors. These regressors comprised of a set of five temporal basis functions (neural mass decays) and a constant term. These decay functions were selected to account for a broad range of temporal trajectories that represent information over multiple time scales (Kiebel et al., 2008). For instance, visual and auditory information change rapidly, whereas the character development, emotional narratives, and so forth of the movie evolve gradually. We derived a model space with all possible combinations of the five temporal basis functions (with the constant term always included). Among a total of 32 models comprising a combination of these basis functions, we identified the winning model using Bayesian model comparison (spm_dcm_bmc_peb.m). A probability of 90% or higher was taken as strong evidence for a winning model. Model 16 had a winning probability of 100%, which incorporated the constant terms and one basis function. To obtain the most representative model at the group level, we pooled the data across all participants.
2. *PEB within participants using the model with winning combinations of temporal basis functions*. To explain the temporal variability of connectivity parameters at subject level, we next used the winning basis functions to individually model single-subject DCM connectivity parameters. Thus, each regressor was associated with eight connectivity parameters. Bayesian model reduction was then employed to identify most consistent between-window single-subject connectivity parameters associated with each “decay” regressor. Regressors that did not contribute to model evidence were pruned away (spm_dcm_peb_bmc.m). The output of this analysis provided the second-level model parameters (i.e., posterior means and covariances).
3. *PEB of PEBs: Group-PEB*. Finally, to know which between-window effects of specific connections were conserved over participants, we performed a second PEB. The second-level parameter estimates (i.e., subject-specific parameters of between-window effects for each DCM estimate) were entered into a group-PEB analysis with a design matrix containing only a constant term. This essentially meant that only average between-window effects across participants were modelled (spm_dcm_bmc_peb.m). Finally, Bayesian model reduction was employed, and any parameters of the group-PEB that did not contribute to the log evidence were pruned away using a greedy search (spm_dcm_peb_bmc.m).

DCM and PEB analyses were also undertaken for the resting-state data shown in Supporting Information Figure S1. DCM and PEB analysis were undertaken with SPM12 opensource toolbox (Ashburner et al., 2014).

### Correlation Analysis

We used the connectivity parameters (DCM estimates) corresponding to feedforward and feedback connections showing consistent between-windows and across-subject effects (PEB analyses) for Pearson’s correlational analysis with HRV and movie features. Instantaneous correlation coefficients were computed after averaging the signals. Data for DCM and movie features corresponding to HRV output were used for correlational analysis. Although naturalistic stimuli bring greater ecological validity when compared to traditional designs, physiological responses such as HR changes are not as precisely time locked to specific stimulus features but show individual variations in timing. In addition, disentangling regional interactions requires time windows, even if using simple measures such as correlation-based dynamic functional connectivity (Zalesky & Breakspear, 2015). Furthermore, prior literature on emotional narratives demonstrate that spoken sentences, social interactions, emotional experience, and the development of a narrative evolve over relatively long time periods (Sonkusare et al., 2019a, 2019b). Empirical evidence also suggests that higher cortical regions like the ACC are influenced by stimulus-related information accumulating for more than 15 sec (Baldassano et al., 2017; Honey et al., 2012). On the other hand, movie features such as audio and luminance show rapid transitions (∼seconds), albeit in a continuously and coherent evolving narrative. Consequently, to allow for a meaningful comparison between connectivity parameters, movie features, and physiological variables (.1 Hz of HRV frequency corresponding to 10 sec), we smoothed these signals by a moving mean of 10 data points (10 sec). The connectivity parameters corresponding to the movie segmentations were serially concatenated and averaged for correlation analyses to find their associations with movies features and HRV. Multiple comparisons were accommodated using a false discovery rate correction (Nichols & Hayasaka, 2003).

## RESULTS

### Power Spectral Features

**Figure 4.**
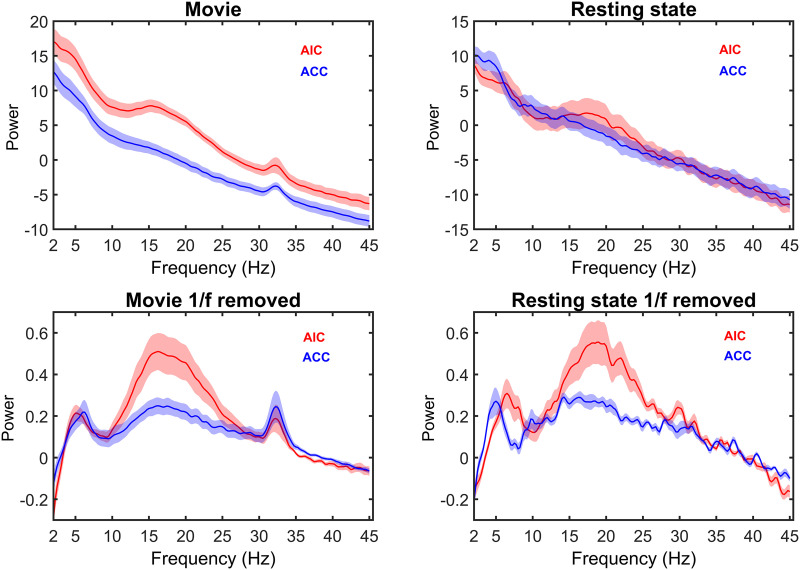
Power spectra. Power profiles of AIC and ACC activity during movie viewing (left) and resting state (right). The bottom row shows the power spectrum after correcting for 1/f power decay. During movie viewing, both the AIC and ACC showed a peak at 30–35 Hz (gamma range). The gamma peak was absent at rest. Shading denotes standard error of mean.

A peak of power in the beta frequency range (14–25 Hz) was observed in the AIC in both the resting and movie viewing data. The ACC and the AIC both show a peak in the gamma range (30–35 Hz) during movie viewing (Figure 4), while this peak was only present in the AIC at rest. Based on these results, we used gamma activity of movie viewing data to investigate functional connectivity between ACC and AIC and its association with movie features. Movie viewing induced highly correlated time-resolved gamma activity between the AIC and the ACC (*r* = .10, *p*_*FDR*_ = 5e^−15^). AIC gamma activity was also correlated positively with the auditory amplitude of the movie (*r* = .10, *p*_*FDR*_ = 1e^−12^). Since there was no gamma peak present in the rest data, functional connectivity was not undertaken for rest data.

### Dynamic Effective Connectivity

**Figure 5.**
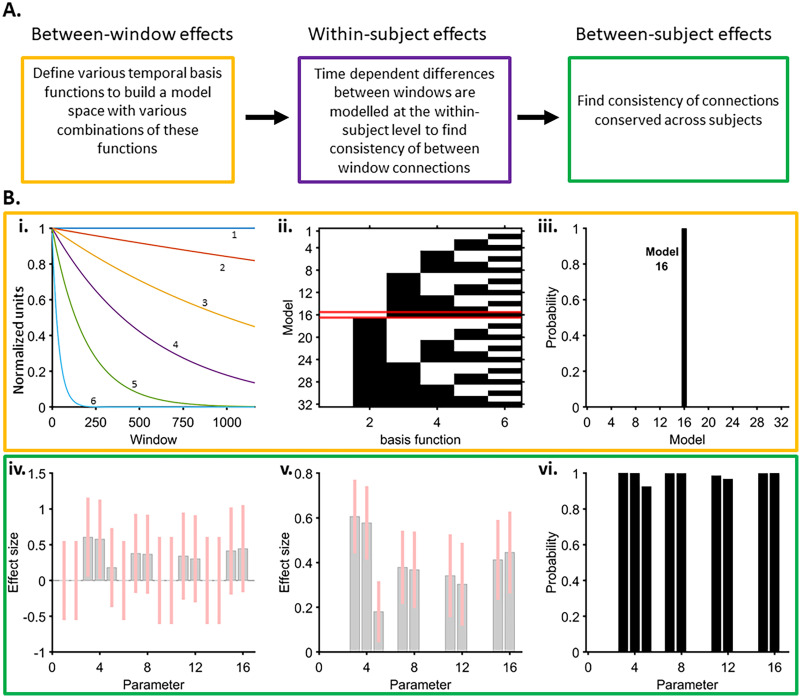
Multilevel parametric empirical Bayes (PEB) analysis of effective connectivity parameters. (A) Overview of the nested PEB analyses. (B i) Regressors [a constant term (1) and five temporal basis functions (2, 3, 4, 5, 6)] considered in a Bayesian general linear model to estimate between-window consistency of effective connectivity parameters. These regressors were selected to cover a wide range of possible neural mass decays. (ii) Model space with all possible combinations of the five temporal basis functions (with the constant term always included). White tiles indicate the presence of temporal basis functions in the model and the black tiles indicates their absence in the model. The winning model (16) is highlighted in red. (iii) Probability of winning model with Bayesian model comparison of all models. The winning model is highlighted in red and comprised of a combination of the constant term and three temporal basis functions (2 in panel A). (iv and v) Group-PEB analysis to identify between-window effects of specific connections conserved over participants without (iv) and with (v) greedy search, respectively. The plots show the connectivity parameters corresponding to models with the constant term (parameters 1–8), and each of the one winning temporal basis function (parameters 9–16) and the related effect sizes. Pink bars denote 90% Bayesian confidence intervals. (vi) Parameters reaching a posterior probability of >90% included feedforward connections (parameters 3, 4, 11, 12) and feedback connections (parameters 5, 7, 8, 15, 16). Only these connectivity parameters were used for further analysis.

After observing gamma-band functional connectivity between AIC and ACC, we estimated the best combination of temporal basis functions that capture the temporal variability (between-window consistency) of effective connectivity. Note than DCM-CSD employed the whole power spectrum from 2 to 45 Hz, thus also incorporating lower frequency modulations. The winning model included a constant term and three temporal basis functions (Figure 5Bi) with >99.9% probability (Figure 5Bii and iii), which was used to model single-subject DCM parameters. A subsequent group-wise PEB analysis to identify the consistency of between-window effects across the participants showed that model parameters incorporating both feedforward and backward connections reached a posterior probability of >99.9% (Figure 5Biv–vi). These connectivity parameters were correlated with the movie features and HRV.

### Feedforward and Feedback Connectivity Parameters Between the AIC and the ACC Are Positively Correlated

We observed several significant relationships between connectivity parameters, HRV, and movie features (Figure 5 and Supporting Information Figure S2). The feedforward connectivity parameters between AIC and ACC showed an inverse relationship (*r* = .13, *p*_*FDR*_ = 8e^−5^), and feedback connectivity also showed a positive correlation (*r* = .13, *p*_*FDR*_ = 4e^−6^). Note that weaker correlations (*r* < .1) are unlikely to be meaningful. However, for completeness these are shown in Supporting Information Figure S2. We also undertook DCM and PEB analyses for resting-state data. We found similar connections that showed between-window and between-subjects consistency (Supporting Information Figure S1). Correlation analyses undertaken for these connectivity parameters did not yield any significant associations (feedforward connectivity of AIC and that of ACC (*r* = .1, *p* = .59), feedback connectivity of AIC and that of ACC (*r* = −.32, *p* = .09), feedforward connectivity of AIC and feedback connectivity from ACC (*r* = −.28, *p* = .14), feedforward connectivity of ACC and feedback connectivity from AIC (*r* = −.25, *p* = .19)).

### Association of AIC-ACC Connectivity With Physiological State and Movie Features

#### HRV.

**Figure 6.**
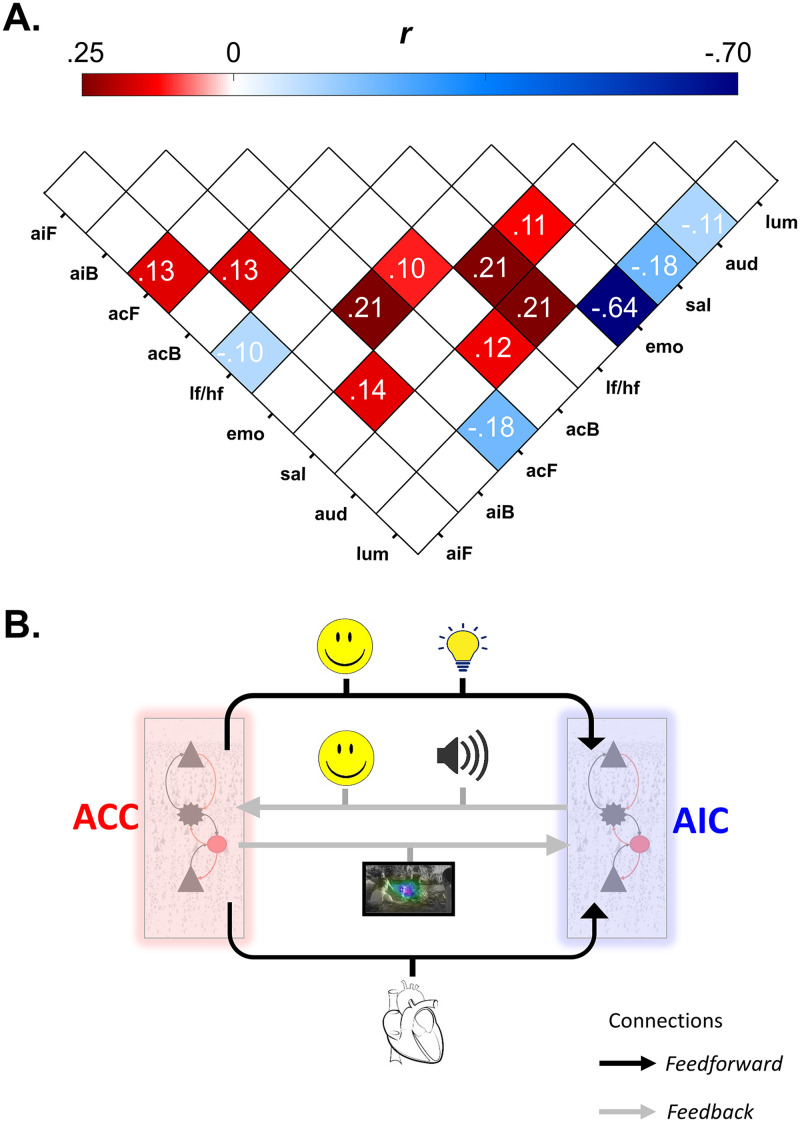
Relationship between effective connectivity, heart rate variability (HRV), and movie features. (A) Pearson’s correlation coefficient values (*r*) between connectivity parameters, HRV and movie features. aiF = feedforward connections from the AIC to the ACC; aiB = feedback connections from the ACC to the AIC; acF = feedforward connections from ACC to AIC; acB = feedback connections from the AIC to the ACC; lf/hf = ratio of low-frequency/high-frequency HRV; emo = emotion scores; sal = salience; aud = audio; lum = luminance. Correlation coefficient values greater than or equal to .10 that survive multiple comparison correction (FDR) with a type I error probability of less than 5% are shown. See Supporting Information Figure S2 for all the correlation and probability values. See Supporting Information Figure S3 for effects of 8- and 10-sec smoothing on correlation results (B) Simplified illustration of feedforward and feedback connectivity between AIC and ACC and their association with data features. Connectivity parameters from the ACC linked to stimuli-related sensory information, whereas AIC-driven connectivity was associated with both the stimulus properties as well as physiological responses. Neuronal cells shown in panel B are adapted from Moran et al. (2013).

Feedforward connectivity from AIC to ACC showed negative correlation with LF/HF ratio (*r* = −.10, *p*_*FDR*_ = .001) (Figure 6A and B).

#### Movie features.

Similarly, feedforward connectivity from ACC to AIC showed positive correlation with facial emotion in the movie (*r* = .21, *p*_*FDR*_ = 5e^−13^) and a negative correlation with luminance (*r* = −.18, *p*_*FDR*_ = 8e^−10^). Feedback connectivity of AIC (connection from ACC) showed positive correlation with movie salience (*r* = .14, *p*_*FDR*_ = 1e^−5^). Similarly feedback connectivity of ACC (connection from AIC) showed significant positive correlation with facial emotion in the movie (*r* = .10, *p*_*FDR*_ = .001) and audio (*r* = .12, *p*_*FDR*_ = 4e^−5^) (Figure 6A and B).

In sum, connectivity from AIC (feedforward and feedback) associated with HRV as well as movie features (emotion and audio) while connectivity from ACC (feedforward and feedback) was associated with emotion, salience, as well as luminance (Figure 6B).

### Associations Between Movie Features and HRV

HRV showed a significant positive correlation with salience (*r* = .21, *p*_*FDR*_ = 1e^−12^) and audio (*r* = .21, *p*_*FDR*_ = 2e^−13^). Emotion scores showed a significant negative correlation with luminance (*r* = −.64, *p*_*FDR*_ = 4e^−135^) a significant positive correlation with salience (*r* = .11, *p*_*FDR*_ = 0.0002).

## DISCUSSION

We study how the dynamic interplay between the ACC and the AIC supports the association between multisensory brain and body responses. We found enhanced gamma neural activity (30–35 Hz) in the ACC and the AIC during movie viewing, but not at rest, is strongly correlated, suggesting a tight coupling between these two regions. Biophysical modelling extended these findings by showing movie-specific patterns of effective connectivity between the ACC and the AIC that link with sensory and somatic signals. Specifically, connectivity parameters from the ACC linked to stimuli-related sensory information, whereas AIC-driven connectivity was associated with both the stimulus properties as well as the physiological responses. These results highlight the distinct, yet complementary, function of the dynamic neural coupling between the AIC and the ACC to process sensory and body signals during a rich and dynamic emotional experience.

Earlier studies mapped the power spectral profile of resting-state iEEG in both the AIC and ACC activity, highlighting a beta peak in the AIC (Frauscher et al., 2018). We replicated these findings and further showed that movie viewing induced gamma (30–35 Hz) activity in both the ACC and the AIC. Similar activity has been detected during discrete task performance, suggesting that gamma activity in the AIC-ACC circuit is ubiquitous to the active process of integrating multisensory information (Bastin et al., 2016; Boroujeni et al., 2021).

Our connectivity findings are in line with the prevailing functional profile attributed to the ACC (Craig, 2009; Critchley et al., 2004). Specifically, we found that feedback connectivity from the ACC to the AIC is linked to external sensory information, in agreement with the notion that the ACC supports the contextual appraisal of incoming sensory information (Harrison et al., 2015). With its extensive patterns of anatomical and functional connectivity with brain regions across all levels of the cortical hierarchy, the ACC seems optimally positioned to compute estimates of stimuli-driven contextual changes (Craig, 2009; Fan et al., 2008; Medford & Critchley, 2010; Valentini, 2010). Accordingly, a number of neuroimaging studies have provided support for such a role of ACC (Luu & Pederson, 2004; Maier & di Pellegrino, 2012). Our result of connectivity from ACC to AIC related to salience, emotion, and luminance potentially highlights the rapid adaptation of ACC activity to changing external properties. The ability to provide context to sensory information underpins core brain functions that have been commonly ascribed to the ACC. These functions include error monitoring (Holroyd & Coles, 2002; Ito et al., 2003; Ullsperger & von Cramon, 2001), cognitive control (Cocchi et al., 2012, 2013; Dosenbach et al., 2006), emotional regulation (Gotlib et al., 2005; Ramirez-Mahaluf et al., 2018), and generating and updating autonomic responses (Chang et al., 2013; Critchley et al., 2003).

Time-resolved AIC connectivity parameters correlated with both emotion, audio and HRV (Figure 4B), suggesting how neural signals generated by the AIC could likely convey both exteroceptive and bodily physiological information. Previous neuroimaging findings support this integrative role of the AIC (Critchley et al., 2005). Specifically, Nguyen et al. (2016) showed that the AIC integrates exteroceptive audio signals encoded by the superior temporal gyrus with interoceptive signals (HRV) processed by the posterior insula. Moreover, it has been shown that respiratory training (via mindfulness) modulated AIC activity evoked by visual task demands (Farb et al., 2013), highlighting a core integratory role of AIC.

Our findings are derived from the canonical microcircuit CMC DCM model, which has been interpreted within a predictive coding framework. Herein, feedforward and feedback connectivity carry prediction error and prediction signals, respectively (Bastos et al., 2012). Our results suggest that predictions and prediction error’s conveyed between the AIC-ACC dyad are positively correlated. These findings support the existence of a synergism underlying the dynamic refining of the link between sensory stimuli properties and physiological processes within the AIC-ACC system. Furthermore, connectivity from ACC to AIC, and its association with exteroceptive movie properties (salience, audio, and luminance), point to a key role of the ACC in continuously monitoring the fluctuating external environment. Feedback connectivity from ACC positively correlated with changes in visual stimuli salience, but feedforward connectivity from ACC negatively correlated with luminance. In this context, and as alluded to earlier about the role of ACC in generating autonomic responses, the functional dissociation is in line with the notion that salient stimuli processing requires sympathetic control (i.e., pupil dilation; Kucyi & Parvizi, 2020), whereas the perceptual adaptation to stimulus’ luminance is under parasympathetic control (i.e., pupil constriction; Szabadi, 2018; Hess & Polt, 1960). On the other hand, feedforward connectivity from AIC negatively correlated with a proxy for sympatho-vagal balance (LF/HF ratio), suggesting the accumulation of predictions errors and the need to update the internal homeostatic status (Owens et al., 2018). Together, the observed patterns of connectivity and associations with stimulus properties imply that iterative computations within the AIC-ACC system play a key role in minimising the overall surprise to the ongoing stream of information. More generally, the casting of our findings within the predictive coding framework extends knowledge on the neural mechanisms linking exteroceptive sensory and body responses (Seth, 2013).

Several limitations need to be considered when interpreting our findings. Despite the richness and quality of the data, it comes from patients with epilepsy. Epilepsy is increasingly seen as a network disorder affecting multiple regions (Bernhardt et al., 2015; Kanner et al., 2017). Some patients had epileptic zones in structurally connected region (e.g., amygdala) or homologous contralateral regions. Although we undertook extensive quality control, residual epilepsy-related artefacts and or pathological activity propagating to the AIC or ACC may nonetheless influence movie-related AIC-ACC circuit activity. Furthermore, the results are based on five individuals in the movie condition. Stereo-EEG clinics remain relatively rare, and access to data for nonclinical research is challenging. In this study, colocation of electrode channels at two regions of interest further constrained the sample size. With unique implantation schemes for each patient, large sample sizes will be required to replicate and extend our findings. In addition, slight variations in the contact locations could also influence the results. However, our results are based on contacts located in relatively small and spatially constrained cortical regions that have been previously demonstrated to serve similar functions (Kolling et al., 2016; Medford & Critchley, 2010; Tian et al., 2019). A granular coverage at the millimetre scale is needed to explore possible spatial specificity of AIC and ACC connectivity.

Another limitation is that most of the contacts were located on the right hemisphere, with only one patient contributing contacts from the left hemisphere. Structural and functional asymmetry of AIC and ACC have been reported (Biduła & Króliczak, 2015; Chiarello et al., 2016; Yan et al., 2009) and thus, further research is needed to explore the possible functional differences of left and right AIC-ACC activity and connectivity. For our effective connectivity analyses, we used the CMC biophysical model. While this model incorporates the general organisation of cortical circuitry (Felleman & Van Essen, 1991), specific differences in the cytoarchitecture of AIC and ACC may affect the quantification of connectivity parameters.

Concerning the temporal trajectories employed in our PEB analyses, each connectivity parameter’s time course was modelled by a linear combination of six temporal basis functions. Although these linear combinations cover a wide range of plausible temporal trajectories, we cannot exclude that more complex (e.g., sinusoidal) trajectories capture additional movie-induced neural dynamics within the AIC-ACC system. We also choose a long smoothing window for our correlational analyses to capture the long temporal scales of high-order associative regions including the ACC and AI (Honey et al., 2012) as well as the slow nature of HRV (∼0.1 Hz). Supplementary analysis did, however, show that results were robust to different smoothing window lengths of 8 and 12 sec (Supporting Information Figure S3).

We also note that the subjective engagement during movie viewing could not be quantified due to the time constraints in the surgical unit. However, we did ensure that participants remained vigilant while watching the movie by continuous monitoring via a wall-mounted camera. Moreover, the quantification of emotional profile of stimulus was based on five negative and one positive valence categories of facial expressions of actors in the movie. Thus, we were unable to investigate possible idiosyncrasies supporting the processing of stimuli with positive and negative emotional valence. We additionally used automated facial emotion expression. Supervised learning algorithms classifying basic facial expressions based on feature values were previously variable but has recently achieved accuracies of 75–98% when benchmarked against manually coded datasets of both posed and spontaneous expressions (Ekundayo & Viriri, 2019). Finally, future studies are required to expand on our analyses by incorporating a wider repertoire of bodily signals, including galvanic skin response and facial expressions.

The characterisation of neural dynamics within the AIC-ACC system has important implications for the study of brain disorders (Menon, 2011). For example, several neuroimaging studies have demonstrated exacerbated stimuli-induced activity and connectivity between the AIC and the ACC in anxiety disorders and obsessive-compulsive disorders (Cocchi et al., 2012; Paulus & Stein, 2006, 2010; Peterson et al., 2014; Pujol et al., 1999; Simmons et al., 2013). Our findings provide new testable hypotheses on the nature of these deficits that may facilitate the development of new targeted therapeutic interventions.

## ACKNOWLEDGMENTS

We thank Annett Koenig for her assistance in arrangements for data acquisition. We thank Kartik Iyer and Caitlin Hall for helpful discussions.

## SUPPORTING INFORMATION

Supporting information for this article is available at https://doi.org/10.1162/netn_a_00295. The data for this project were acquired from clinical epilepsy participants undergoing clinical care and consenting for additional research protocols. Local ethics approval mandated strict privacy restrictions around their availability outside of the named investigator team. Researchers wishing to access these data will require local ethics approval and a data sharing agreement with QIMR Berghofer and Mater Hospital Brisbane.

## AUTHOR CONTRIBUTIONS

Saurabh Sonkusare: Conceptualization; Data curation; Formal analysis; Investigation; Methodology; Project administration; Resources; Software; Visualization; Writing – original draft; Writing – review & editing. Katharina Wegner: Formal analysis; Methodology; Writing – review & editing. Catie Chang: Methodology; Supervision; Writing – review & editing. Sasha Dionisio: Methodology; Resources; Writing – review & editing. Michael Breakspear: Conceptualization; Funding acquisition; Investigation; Project administration; Supervision; Writing – original draft; Writing – review & editing. Luca Cocchi: Conceptualization; Funding acquisition; Supervision; Writing – original draft; Writing – review & editing.

## FUNDING INFORMATION

Michael Breakspear, QIMR Berghofer Medical Research Institute (https://dx.doi.org/10.13039/100013103), Award ID: 6626. Luca Cocchi, Australian National Health Medical Research Council, Award ID: GN2001283.

## TECHNICAL TERMS

Interoception: Registration, interpretation, and integration of sensory signals from within the body.
Heart rate variability: Fluctuation in durations between successive heart beats.
Effective connectivity: Causal influence that a brain region exerts over another region.
Dynamic causal modelling: Computational framework to infer effective connectivity.
Exteroception: Registration, interpretation and integration of sensory signals from outside the body (external environment).
Parametric empirical Bayes (PEB): A Bayesian framework to infer commonalities and differences in the data.
