# Supplementary material for: Dynamic interactions between anterior insula and anterior cingulate cortex link perceptual features and heart rate variability during movie viewing
Source: Netw Neurosci. 2023 Jun 30;7(2):557–77. doi: 10.1162/netn_a_00295 (PMC10312263; doi:10.1162/netn_a_00295)
Supplement: Supplementary file 1 [file netn-7-2-557-s001.pdf]

Sonkusare, S., Wegner, K., Chang, C., Dionisio, S., Breakspear, M. & Cocchi, L. (2022). Supporting information for “Dynamic interactions between anterior insula and anterior cingulate cortex link perceptual features and heart rate variability during movie viewing.” *Network Neuroscience*. Advance publication. https://doi.org/10.1162/netn_a_00295

Supplementary Materials

Dynamic interactions between anterior insula and anterior cingulate cortex link perceptual features and heart rate variability during movie viewing

Saurabh Sonkusare, Katharina Wegner, Catie Chang, Sasha Dionisio, Michael Breakspear, Luca Cocchi

Sonkusare, S., Wegner, K., Chang, C., Dionisio, S., Breakspear, M. & Cocchi, L. (2022). Supporting information for "Dynamic interactions between anterior insula and anterior cingulate cortex link perceptual features and heart rate variability during movie viewing." *Network Neuroscience*. Advance publication. https://doi.org/10.1162/netn_a_00295

Supplementary Table 1: Participant profile and MNI coordinates of contacts from resting state data

**Participant ID**	**Gender**	**Age (yrs)**	**AIC MNI coordinates (x,y,z)**	**ACC MNI coordinates (x,y,z)**
P6	M	32	37, 16, 1<br>36, 19, 5	3, 32, -8<br>8, 32, -8
P30	F	32	32, 15, 1<br>31, 16, 5	4, 32 ,-16.5<br>8, 33, -17
P50	M	35	30, 24, -10<br>33, 24, -7	-10, 38, 20
P60	M	38	28, 24, -8<br>31, 25, -7<br>34, 25, -4.5	-5, 29, 25
P76	M	38	-25, 25, -3<br>-31, 25, -3<br>-37, 26, -1	-13, 24, -5.5<br>-19, 24, -4.5
P106	M	27	38, 0.5, 1	9, 28.1, -11.5

Sonkusare, S., Wegner, K., Chang, C., Dionisio, S., Breakspear, M. & Cocchi, L. (2022). Supporting information for “Dynamic interactions between anterior insula and anterior cingulate cortex link perceptual features and heart rate variability during movie viewing.” *Network Neuroscience*. Advance publication. https://doi.org/10.1162/netn_a_00295

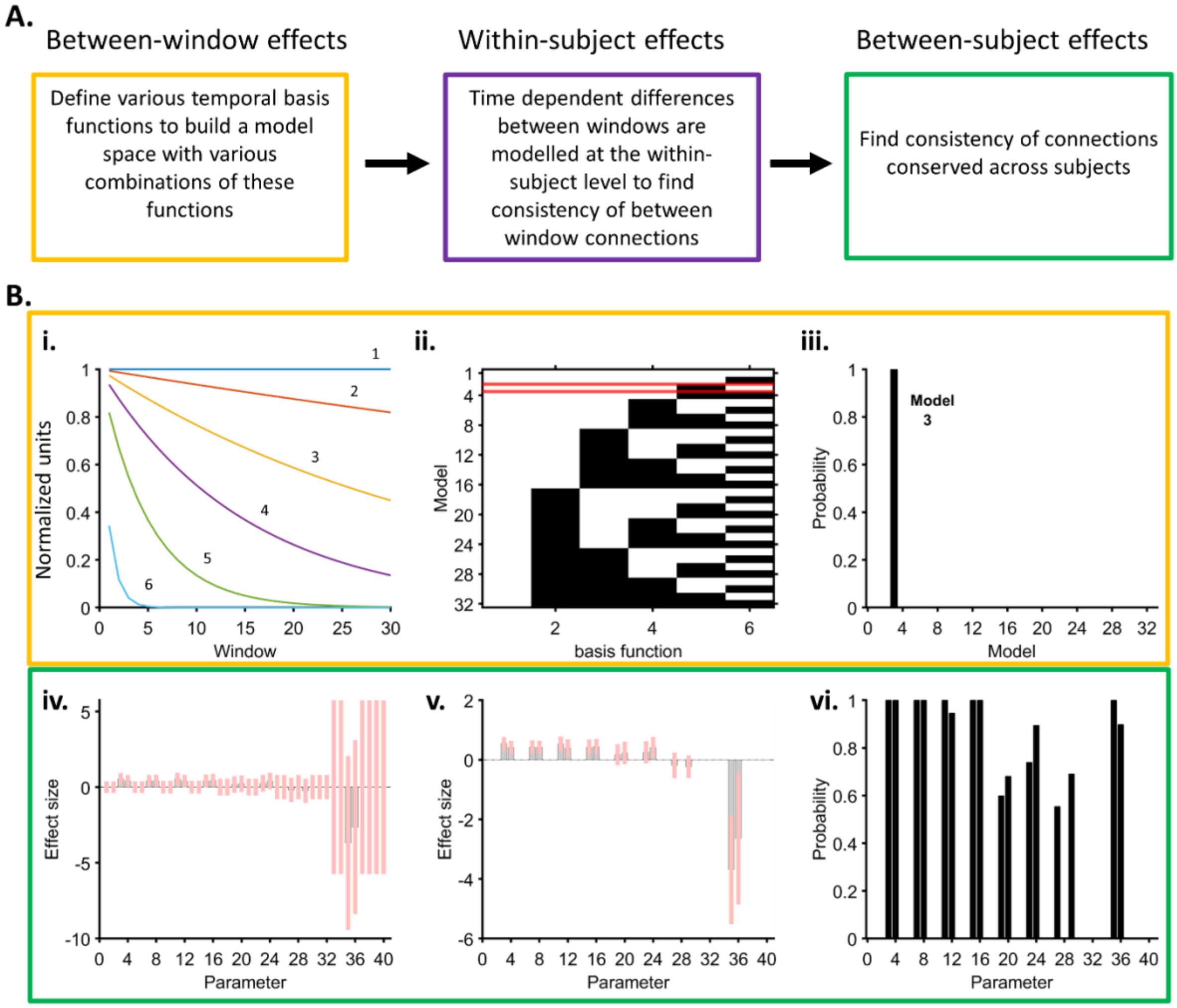

Supplementary Figure 1. *Multi-level Parametric Empirical Bayes (PEB) analysis of effective connectivity parameters from resting state data.* **A:** Overview of the different PEB analyses. **B i:** Regressors [a constant term (1) and five temporal basis functions (2,3,4,5,6)] considered in a Bayesian general linear model to estimate between-window consistency of effective connectivity parameters. These regressors were selected to cover a wide range of possible neural mass decays. **ii:** Model space with all possible combinations of the five temporal basis functions (with the constant term always included). White tiles indicate the presence of temporal basis functions in the model and the black tiles indicates their absence in the model. The winning model (3) is highlighted in red. **iii:** Probability of winning model with Bayesian model comparison of all models. The winning model 3 comprised of a combination of the constant term and three temporal basis functions (2,3,4,6 in panel A). **iv & v:** Group-PEB analysis to identify between-window effects of specific connections conserved over participants without (iv) and with (v) greedy search, respectively. The plots show the connectivity parameters corresponding to models with the constant term (parameters 1-8) and each of the one winning temporal basis function (parameters 9-40) and the related effect sizes. Pink bars denote 90% Bayesian confidence intervals. **vi:** Parameters reaching a posterior

Sonkusare, S., Wegner, K., Chang, C., Dionisio, S., Breakspear, M. & Cocchi, L. (2022). Supporting information for “Dynamic interactions between anterior insula and anterior cingulate cortex link perceptual features and heart rate variability during movie viewing.” *Network Neuroscience*. Advance publication. https://doi.org/10.1162/netn_a_00295

probability of >90% included feedforward connections (parameters 3, 4, 11, 12, 35, 36) and feedback connections (parameters 7, 8, 15, 16). These connections are consistent with the connections preserved from movie watching data.

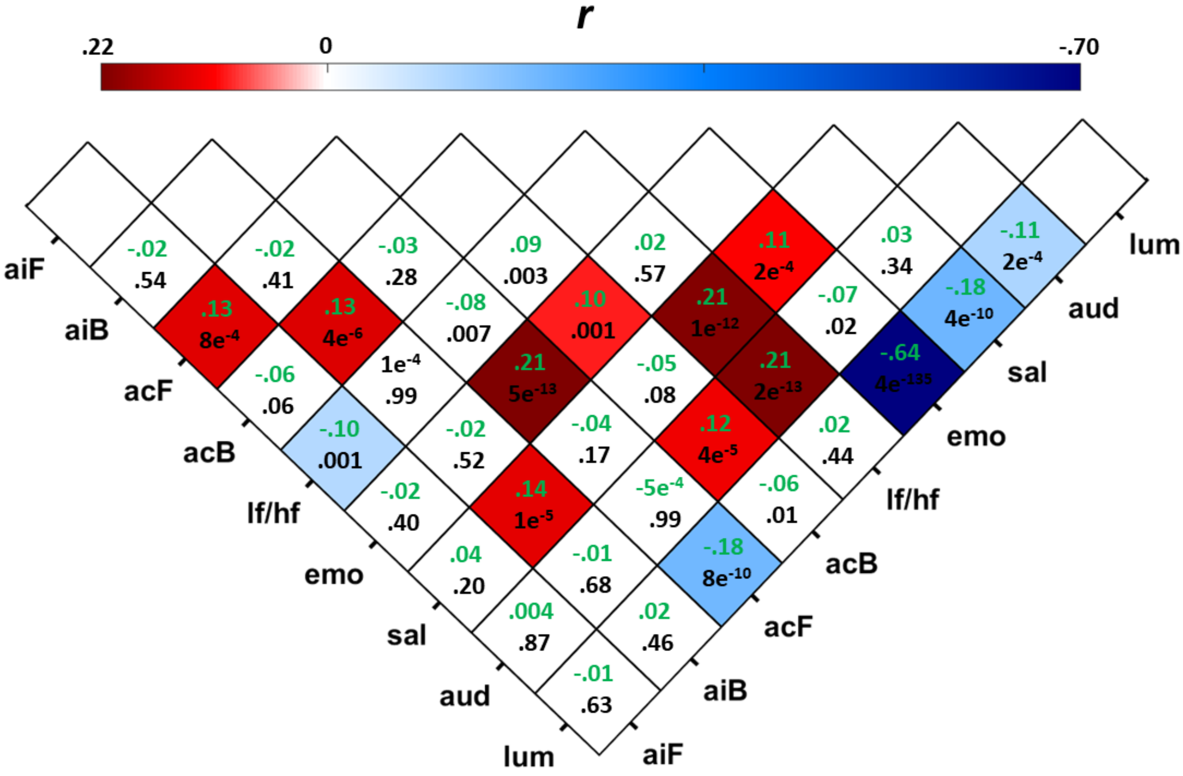

Supplementary Figure 2. *Relationship between effective connectivity, heart rate variability (HRV), and movie features*. Each tile shows Pearson’s correlation coefficient values (r) in green and corresponding p-values in black between connectivity parameters, HRV, and movie features. aiF = feedforward connections from the AIC to the ACC, aiB = feedback connections from the ACC to the AIC, acF = feedforward connections from ACC to AIC, acB = feedback connections from the AIC to the ACC, lf/hf = ratio of low frequency/high frequency HRV, emo – emotion scores, sal – salience, aud – audio, lum – luminance.

Sonkusare, S., Wegner, K., Chang, C., Dionisio, S., Breakspear, M. & Cocchi, L. (2022). Supporting information for "Dynamic interactions between anterior insula and anterior cingulate cortex link perceptual features and heart rate variability during movie viewing." *Network Neuroscience*. Advance publication. https://doi.org/10.1162/netn_a_00295

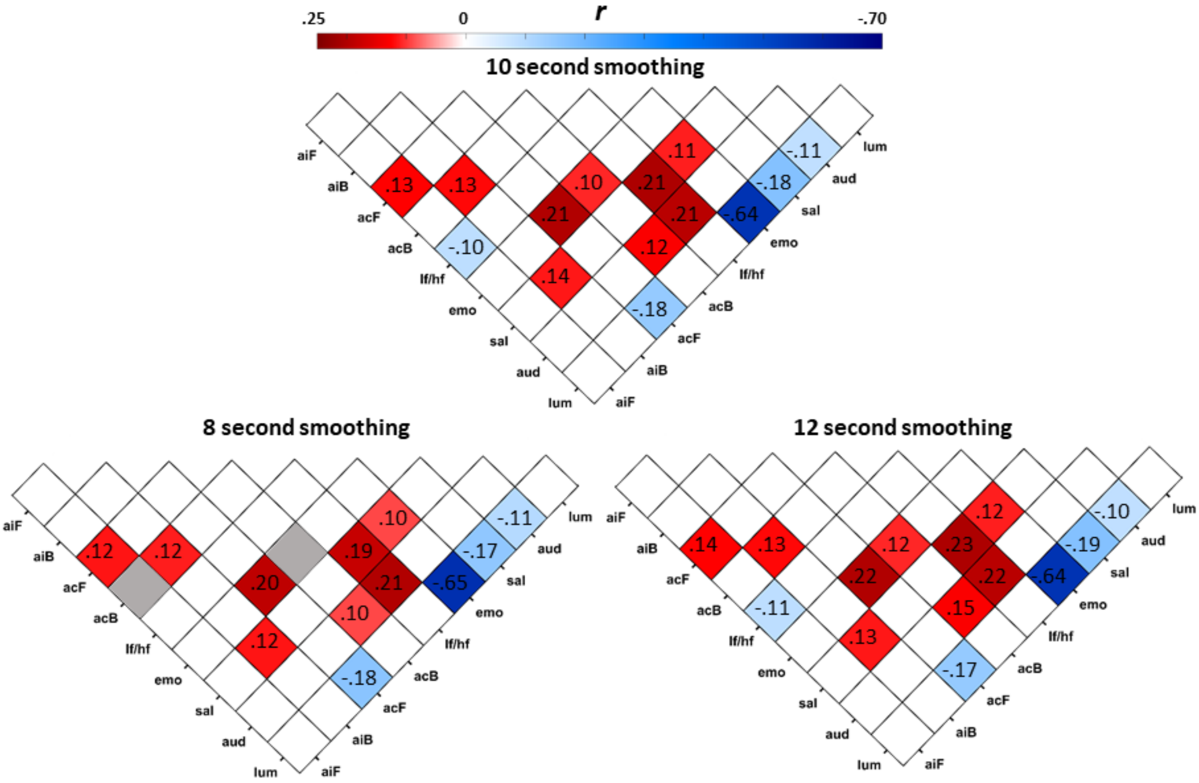

Supplementary Figure 3. Effect of different windows on correlational analyses between effective connectivity, heart rate variability (HRV), and movie features. Top plot shows the results with 10second smoothing. Bottom left plot shows the significant (surviving FDR correction) associations with 8 second smoothing whilst the bottom right plot shows the significant associations with 12 second smoothing. Coloured tiles shows Pearson's correlation coefficient values (r) in black. Correlations that are no longer statistically significant with 8 second smoothing are shown in grey. aiF = feedforward connections from the AIC to the ACC, aiB = feedback connections from the ACC to the AIC, acF = feedforward connections from ACC to AIC, acB = feedback connections from the AIC to the ACC, lf/hf = ratio of low frequency/high frequency HRV, emo – emotion scores, sal – salience, aud – audio, lum – luminance.
